# Obsessive–compulsive symptoms in a large population-based twin-family sample are predicted by clinically based polygenic scores and by genome-wide SNPs

**DOI:** 10.1038/tp.2015.223

**Published:** 2016-02-09

**Authors:** A den Braber, N R Zilhão, I O Fedko, J-J Hottenga, R Pool, D J A Smit, D C Cath, D I Boomsma

**Affiliations:** 1 Department of Biological Psychology, VU University Amsterdam, Amsterdam, The Netherlands; 2 Alzheimer Center & Department of Neurology, VU University Medical Center and Neuroscience Campus, Amsterdam, The Netherlands; 3 Department of Clinical and Health Psychology, Utrecht University, Utrecht, The Netherlands; 4 Altrecht Academic Anxiety Disorders Center, Utrecht, The Netherlands

## Abstract

Variation in obsessive–compulsive symptoms (OCS) has a heritable basis, with genetic association studies starting to yield the first suggestive findings. We contribute to insights into the genetic basis of OCS by performing an extensive series of genetic analyses in a homogeneous, population-based sample from the Netherlands. First, phenotypic and genetic longitudinal correlations over a 6-year period were estimated by modeling OCS data from twins and siblings. Second, polygenic risk scores (PRS) for 6931 subjects with genotype and OCS data were calculated based on meta-analysis results from IOCDF-GC, to investigate their predictive value. Third, the contribution of measured single nucleotide polymorphisms (SNPs) to the heritability was estimated using random-effects modeling. Last, we performed an exploratory genome-wide association study (GWAS) of OCS, testing for SNP- and for gene-based associations. Stability in OCS (test–retest correlation 0.63) was mainly explained by genetic stability. The PRS based on clinical samples predicted OCS in our population-based twin-family sample. SNP-based heritability was estimated at 14%. GWAS revealed one SNP (rs8100480), located within the MEF2BNB gene, associated with OCS (*P*=2.56 × 10^−8^). Additional gene-based testing resulted in four significantly associated genes, which are located in the same chromosomal region on chromosome 19p13.11: MEF2BNB, RFXANK, MEF2BNB-MEF2B and MEF2B. Thus, common genetic variants explained a significant proportion of OCS trait variation. Genes significantly associated with OCS are expressed in the brain and involved in development and control of immune system functions (RFXANK) and regulation of gene expression of muscle-specific genes (MEF2BNB). MEF2BNB also showed a suggestive association with OCD in an independent case–control study, suggesting a role for this gene in the development of OCS.

## Introduction

Obsessive–compulsive disorder (OCD) is a neuropsychiatric disorder characterized by recurrent, persistent and intrusive anxiety-provoking thoughts or images (obsessions) and subsequent repetitive behaviors (compulsions).^1^ The lifetime prevalence of OCD has been estimated between 0.5 and 2.0%,^1^ and among all anxiety disorders, it is known as a major cause of social impairment and a leading cause of non-fatal disease burden worldwide.^2^

It is clear that genetic factors are important in the etiology of obsessive–compulsive symptoms (OCS), with heritability estimated ~40%.^3, 4^ These results did not vary with sex or symptom severity.^3^ Consistent with what is expected for individual single nucleotide polymorphism (SNP) effect sizes in highly polygenic traits, the first molecular association studies for OCD have not identified large effect variants.^5, 6, 7^ Taylor *et al.*^7^ performed a comprehensive meta-analysis of genetic association studies in OCD, including 113 relevant studies. Their main meta-analysis showed that OCD was associated with serotonin-related polymorphisms (5-HTTLPR and HTR2A) and, in males only, with polymorphisms involved in catecholamine modulation (COMT and MAOA). A secondary meta-analysis by the same group, which targeted polymorphisms that were investigated in fewer than 5 datasets, identified another 18 polymorphisms with significant odds ratios. These polymorphisms might be useful candidates for further investigation, although most results were based on candidate gene studies and must be treated with care due to the possible confounding effects of population stratification.

The international OCD foundation Genetics Collaborative (IOCDF-GC; Stewart *et al.*),^5, 7^ conducted an ancestry-stratified case–control genome-wide association (GWA) analysis, in 1465 cases, 5557 ancestry-matched controls and 400 trios, consisting of 1 affected offspring with 2 parents. The trio analysis revealed a significant SNP near the BTBD3 gene (rs6131295). However, this variant lost genome-wide significance when meta-analyzed with the case–control data. The meta-analysis showed a significant enrichment of methylation quantitative trait loci (*P*<0.001) and frontal lobe expression for the top-ranked SNPs (*P*<0.01).^5^ Recently, a second multinational collaboration (OCD Collaborative Genetics Association Study (OCGAS); Mattheisen *et al.*).^8^ performed a GWA study (GWAS) in 1598 patients and 3473 controls. The smallest *P*-value was observed for a marker near the PTPRD gene on chromosome 9 with *P*=4.13 × 10^−^^7^.^8^ Gene-based testing for associations at the gene instead of SNP level, revealed a significant association of IQCK, C16orf88 and OFC11.^8^ These findings await replication.

With this study, we aim to gain further insights into the genetic basis of OCS by performing a series of analyses in a homogeneous, population-based sample of twin families, registered with the Netherlands Twin Registry (NTR). Well-phenotyped population cohorts may contribute to the understanding of the architecture of common complex disorders. We first performed genetic structural equation modeling (SEM)^9^ to estimate twin–twin and twin–sibling correlations and heritability for OCS in adults as measured with the Padua Inventory Revised Abbreviated (PI-R-ABBR).^10^ Information on the PI-R-ABBR was available for two time points (2002 and 2008), which allowed calculation of the stability of OCS across a 6-year period. The long-term stability of the phenotype puts an upper limit to heritability—that is, reveals the proportion of total variation across time that is due to differences among individuals, and puts genetic association studies into perspective. Next, we investigated whether polygenic risk scores (PRS) based on a GWA analysis of clinical OCD cases^5^ significantly predict OCS in our population-based sample. If so, this indicates genetic overlap between the two types of samples, and suggests that future GWA studies can benefit from combining both population-based and case–control samples. We estimated the proportion of phenotypic variance explained by all autosomal SNPs using the Genome-wide Complex Trait Analysis (GCTA) software (Brisbane, QLD, Australia) in a sample of related and unrelated individuals. Further, we performed an explorative GWAS on OC symptom scores from 6931 subjects and entered the GWAS output into a gene-based analysis, to test for associations at the gene rather than the single SNP level.^11^

## Materials and methods

### Participants and measures

Study data were collected in participants of the NTR.^12, 13^ We analyzed data collected in 2002 and 2008 using the PI-R-ABBR.^14^ The total sample with phenotype data from either the 2002 and/or 2008 data collection contained 20 376 individuals from 7812 families. All individuals with information from at least one survey were included in the analysis. A total of 10 134 individuals had sum scores available for the PI-R-ABBR collected in 2002 and 15 720 individuals had sum scores for the PI-R-ABBR collected in 2008. Longitudinal data were available for 5478 individuals. The distribution of the OC symptom scores, from the PI-R-ABBR collected in 2002 and 2008, is provided in Supplementary Figure 1.

For more information on the PI-R-ABBR OCS measures, please see Supplementary Methods ‘participants and measures'.

On 6931 subjects (twins, their siblings, parents and spouses), phenotypic and genetic data were available. These subjects were entered into the genetic analyses (PRS, GWAS). For GCTA analysis, from these 6931, 6881 subjects were included in the Genetic Relationship Matrix. If a subject filled out both surveys we choose to enter the score of the last survey into GWAS and GCTA. Table 1 gives an overview of subjects per analysis and their demographics (see also Supplementary Methods ‘Subjects entered into different genetic analyses' for more details and the study by Zilhão *et al.*).^15^

This study has been approved by the Central Ethics Committee on Research involving human subjects of the VU University Amsterdam. Informed Consent was obtained from all subjects.

### Genotyping and imputation

DNA was extracted from either blood or buccal cell samples that were collected for various projects done by the NTR.^12, 13^ A total of 31,265,038 SNPs were included in the analysis using the 1000 Genome phase 1 version 3 as a reference panel. For further details on genotyping, quality control and imputation methods see Supplementary Methods ‘genotyping and imputation'.

### Heritability estimates from SEM

To estimate the total contribution of genetic factors to trait variance and to the longitudinal covariance, the resemblance among twins and siblings was compared. Monozygotic (MZ) twins share (nearly) all their genes, whereas dizygotic (DZ) twins, just like non-twin siblings, share on average half of their genetic variation. In quantitative genetics, this information is used to decompose the total variance of a trait into additive genetic (A), non-additive genetic or dominance (D), and unshared environmental variance (E). The greater the phenotypic similarity between MZ twins, when compared with DZ twins and non-twin siblings, the more of the variance of the trait is attributed to genetic factors. Genetic analyses were carried out by SEM as implemented in OpenMx.^9^ Further details on SEM analysis may be found in Supplementary Methods ‘SEM methods'.

### Polygenic risk scores

To examine shared polygenic risk at an aggregate level between two independent GWAS samples, we used genetic risk score profiling as described by Purcell *et al.*^16^ GWAS results (SNP, effect allele, effect size as represented by a Beta-value and *P*-value) obtained from the analysis in the European case–control sample by Stewart *et al.*^5^ were used as a discovery dataset for calculating PRS within our NTR target sample in Plink. PRS were then regressed against the OCS scores from the NTR dataset (*n*=6931) to calculate the proportion of variance in this target set explained by PRS obtained from the discovery set, with 15 statistical cutoffs for SNP inclusion in the score (cutoffs: *P*<0.00001, *P*<0.0001, *P*<0.001, *P*<0.01, *P*<0.05, *P*<0.1, *P*<0.2, *P*<0.3, *P*<0.4, *P*<0.5, *P*<0.6, *P*<0.7, *P*<0.8, *P*<0.9, *P*⩽1). To correct for family relatedness in the NTR sample, generalized estimating equations in SPSS with the exchangeable and robust function were used.^17^ Sex, age, principal components to correct for the population substructure^18^ and genotyping platform were included as covariates. As an additional test, we regressed the PRS on a non-psychiatric, and OCS-uncorrelated trait (height), from the NTR (*n*=6715); we sought to rule out a possible spurious association consequent of incomplete correction for population stratification and/or cryptic relatedness between the discovery and target datasets. Finally, to test for concordance of effect directions across both datasets, we performed Fisher's exact statistical test using the online-based application SECA (SNP effect concordance analysis)—http://neurogenetics.qimrberghofer.edu.au/SECA/.^19^ For 12 subsets of SNPs with *P*<(0.01, 0.05, 0.1, 0.2, 0.3, 0.4, 0.5, 0.6, 0.7, 0.8, 0.8, 0.9, 1.0) in both datasets, Fisher's exact tests were performed to evaluate if there is an excess of SNPs in the first dataset (European case–control study) with same direction of effect in the NTR dataset across the total 144 SNP subsets. An empirical *P*-value was generated by permutations (1000) for observing the number of SNP subsets with nominally significant concordance.

### Estimations of variance explained by common SNPs (GCTA)

The variance of OCS explained by measured and imputed SNPs was estimated with GCTA using the Restricted Maximum Likelihood procedure.^20, 21^ This method gives insight into the contribution to the additive genetic variance of all genotyped and imputed SNPs. This provides an upper bound on the variance that can be explained by the set of SNPs. To analyze all available data we followed the method proposed by Zaitlen *et al*,^22^ in which the SNP-based and kinship-based heritability can be estimated simultaneously. For further details on the method followed and quality control, refer to Supplementary Methods ‘estimations of variance explained by common SNPs (GCTA)'.

### GWAS

GWA analysis was conducted with linear regression under an additive model with adjustment for age, age-squared, principal components of genetic ancestry, genotyping platform and sex. SNPs with values of *P*<5.00E−08 were declared genome-wide significant. For further details on this analysis, refer to Supplementary Methods ‘GWAS'.

### Gene-based analysis

GATES, as implemented in the open-source tool Knowledge-Based Mining System for Genome-Wide Genetic Studies (KGG, version 3.0, Hong Kong, China), was used to perform a gene-based genome-wide analysis.^11^ GATES employs an extension of the Simes procedure^23^ to assess the significance of a statistical association at the gene level, by combining the individual genotype–phenotype association tests applied at each single marker. In short, it sums all the individual SNP *P*-values, available from GWAS summary data, within a gene to output a gene-based *P*-value. The effective number of independent *P*-values is given by appropriately controlling for the Linkage Disequilibrium structure on the specified SNPs. SNPs were allocated to genes including gene boundaries of ±5 kb from the 5′and 3′ untranslated region. To correct for multiple testing false discovery rate was set at *Q*<0.05.

## Results

### Demographics

For subjects who completed the PI-R-ABBR in 2002 and in 2008 the average age was 33.0 (s.d.=11.5), and 34.7 (s.d.=14.6). Retest stability for OCS scores over a time span of ~6 years was 0.63. The effect of age on OCS scores was a drop of 0.03 per year for both PI-R-ABBR collected in 2002 and 2008.

### Genetic modeling (SEM)

MZ, DZ/sibling, test–retest and cross-correlations are summarized in Table 2. Twin correlations for MZ males and MZ females were equal, as were twin/sibling correlations for DZ males, DZ females, DZ opposite-sex twins and siblings. This indicates that there is no evidence for qualitative sex differences in the heritability of OCS and that to a large extent the same genes influence these symptoms in males and females. Twin correlations were more than twice as large in MZ as compared with DZ/sib pairs, indicating that phenotypic similarity is predominantly accounted for by genetic effects rather than shared environment. The same pattern was observed for cross-twin–cross-time correlations, indicating that the observed stability is also mainly caused by genetic factors. SEM showed a significant heritability (*P*<1.0 × 10^−10^) for OCS at both time points of 0.42 (95% confidence interval (CI) PI-R-ABBR 2002=0.371–0.467 for, and 95% CI PI-R-ABBR 2008 =0.379–0.456). The estimation of the bivariate (broad sense) heritability found that 56% (95% CI=0.497–0.619) of the stability of OC symptom was due to genetic factors (both additive and dominant components), and the longitudinal additive genetic correlation, that is, the degree of overlap between additive genetic influences at both time points was 0.58.

### PRS regressed on PI-R-ABBR sum scores

**Figure 1:**
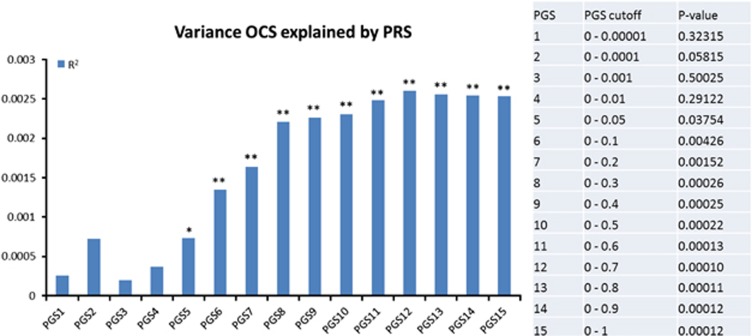
Proportion of variance in OC symptom scores, as measured in the NTR sample, explained by polygenic scores (PRS) obtained from European case–control sample by Stewart *et al.*^5^, with a range of 15 statistical cutoffs for SNP inclusion in the score (PRS1; *P*<0.00001, PRS2; *P*<0.0001, PRS3; *P*<0.001, PRS4; *P*<0.01, PRS5; *P*<0.05, PRS6; *P*<0.1, PRS7; *P*<0.2, PRS8; *P*<0.3, PRS9; *P*<0.4, PRS10; *P*<0.5, PRS11; *P*<0.6, PRS12; *P*<0.7, PRS13; *P*<0.8, PRS14; *P*<0.9, PRS15; *P*⩽1). *P<0.05; **P<0.01. NTR, Netherlands Twin Registry; OC, obsessive–compulsive.

The proportions of variance of OCS in the NTR sample explained by PRS obtained from the discovery set (European case control sample Stewart *et al.*),^5^ using the 15 statistical cutoffs for SNP inclusion in the score, are summarized in Figure 1. Results show that when including more SNPs in the analyses an increasing amount of variance in OCS is explained, reaching a plateau of 0.2% explained variance (*P*<0.001) at PRS12 (includes all SNPs from Stewart *et al.*^5^ with a *P*<0.7 (*N*=4,288,152)). Supplementary Figure 2 provides the results obtained when using height as the outcome phenotype for the target set for the same PRS. For the same 15 statistical cutoffs, no significant result was observed (*P*<0.01), that is, the PRS did not explain any variance in height, hence excluding the confounding effect due to cryptic relatedness across sample sets and possible residual genetic stratification effects present in both populations. Finally, Supplementary Figure 3 presents the results in a heat map plot from analysis of concordance using SECA. The permuted *P*-value for the number of SNP subsets nominally significant was *P*=0.002 thus indicating significant concordance of genetic risk across the datasets.

### SNP-based heritability

GCTA results showed a significant SNP-based heritability estimate of 0.14 (s.e.=0.05, *P*=0.003), and a total narrow-sense heritability of 0.34 (s.e.=0.02, *P*<0.001). Thus 14% of the total phenotypic variance in OCS can be accounted for by the SNPs in the genotyping platform, and the total set of SNPs included in an additive genetic model account for 34% of the total heritability.

### GWAs analysis

**Figure 2:**
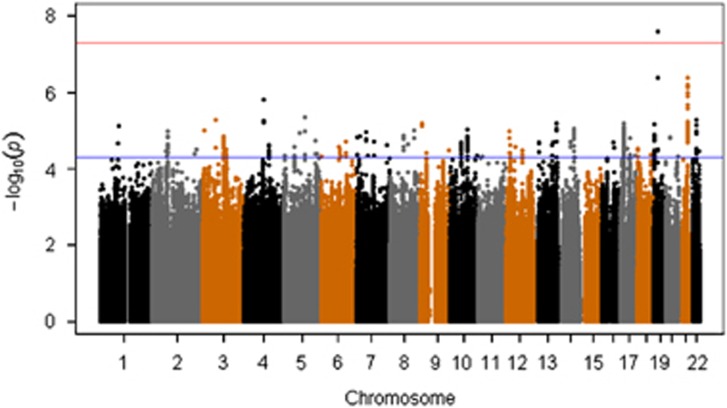
Manhattan plots of all genotyped single nucleotide polymorphisms (SNPs). Red and blue lines indicate significance thresholds of 5.00E−08 and 1.00E−05, respectively.

Top associated variants in GWAS analysis (top 20 SNPs) are summarized in Table 3 (a more comprehensive overview of these results is present in Supplementary Table 1). The Manhattan plot, showing the −log(*P*) plotted against genomic location, and QQ plot of observed versus expected −log(*P*) statistics for the OC symptom GWAS, are illustrated in Figure 2 and Supplementary Figure 4, respectively. Of the top associated variants, one SNP, rs8100480 (19299079, 19p13.11 (hg19), *P*=2.56 × 10^−8^), exceeded the threshold for genome-wide significance and showed a positive association with OCS. This SNP is located within the *MEF2BNB* gene. A more detailed look into this region is provided by the regional association plot in Supplementary Figure 5.

Further, we searched in our results whether they replicate top SNPs reported by Stewart *et al.*^5^ and Mattheisen *et al.*^8^ Supplementary Table 2 summarizes results for the strongest associated GWAS variants from Stewart *et al.*^5^ From their table of 43 SNPs (with *P*<1.0 × 10^−5^), 16 were found to be independent.^8^ None of these SNPs were significantly replicated in our sample when correcting for multiple comparison (0.05/16=0.003). However, a trend (*P*=0.0049) for replication was found for rs4868342, located on chromosome 5. This SNP is located within the HMP19 gene.

Supplementary Table 3 summarizes results for the strongest associated GWAS variants from Mattheisen *et al.*^8^ None of the 32 suggestive associations (with *P*<1.0 × 10^−4^) were replicated in our sample after correcting for multiple comparison (0.05/32=0.001). Neither were the significant results obtained in our study replicated in the Stewart sample (data not shown).

### Gene-based analysis

A total of 2,644,694 SNPs were mapped to 22 759 genes. The QQ plots with the observed versus expected –log(*P*) of the association tests are presented in Supplementary Figure 6. Table 4 depicts all the genes with a significant association. Although these are all nominal significant, the Benjamin–Hochberg procedure was set to control for the *q*<0.05.^23^ After correction, four genes remained significant, the *regulatory factor X-associated ankyrin-containing protein (RFXANK), the myocyte enhancer factor 2B* (*MEF2B*), the *MEF2B neighbor* (*MEF2BNB*) and the *MEF2BNB-MEF2B* read through (*MEF2BNB-MEF2B*). All these genes are located in the same chromosomal region (19p13.11), and share the SNP with the lowest *P*-value annotated to the gene (rs8100480, *P*=2.56E−08). The Manhattan plot for the gene association *P*-values is present in Supplementary Figure 7.

## Discussion

This study aimed at getting a better insight into the genetic basis of OCS in a population-based sample.

First, in line with previous studies, we estimated the heritability for OCS at 0.42.^4^ Stability of OCS over a 6-year time period was 0.63, and cross-twin–cross-time correlations were found to be twice as high in MZ compared with DZ/sib pairs, indicating that the observed stability is mainly caused by genetic factors. Bivariate analyses showed a longitudinal genetic correlation of 0.58. Second, polygenic scores based on a GWA analysis of clinical OCD cases significantly predicted OCS in the independent population-based sample. Analysis of concordance of results indicate that the genetic risk between the two datasets are concordant. These results indicate a genetic overlap between OCD assessed as a categorical disorder and OCS assessed as a continuous trait. Furthermore, the fact that the proportion of variance explained increases as more SNPs are included in the analysis indicates that many small effect variants contribute to the trait. Therefore, future GWA studies can benefit from the inclusion of both population-based and case–control studies, and by analyzing OCD as a quantitative rather than a categorical trait.

Third, we estimated the SNP-based heritability. We were able to find significant explained variance at 0.34. More importantly we were able to partition the heritability into two components, revealing a SNP heritability of 0.14 and the heritability not accounted for by SNPs at ~0.20. These results corroborate well with our findings from our twin model (broad heritability at 0.42). In a previous study by Davis *et al.*^24^ using a case–control design, the heritability of OCD was estimated at 0.37. Although our results do not completely agree, different study-designs can render somewhat different estimates, as was shown by Golan *et al.*^25^

Fourth, the GWAS performed on the continuous OC symptom scores resulted in one significant SNP (rs8100480), which is located in the *MEF2BNB-MEF2B* gene. In addition, we sought to calculate gene-based *P*-values, to determine whether there are genes with associated SNPs, which can collectively achieve statistical significance. Implementing a gene-based analysis as a follow-up complementary analysis is of additional value over traditional GWAS results. Gene-based analysis takes the number of SNPs per gene and gene size into account, considering genes as functional units and informing on the underlying genetic architecture of the phenotype. Furthermore, since for most psychiatric disorders we do not expect large effect causal variants, replication analysis from underpowered GWAS would not reflect these findings. Gene-based tests, by providing an aggregated analysis, may successfully capture and replicate those aggregate effects. We found significant associations for four genes. *RFXANK* encodes a protein that belongs to the MHC class II molecules, which has an important role in the development and control of the immune system. Bare lymphocyte syndrome 2, an immunodeficiency disorder, has been linked with mutations in this gene. Although *RFXANK* has not been identified in OCD previously, several lines of research have indicated a role of immune system alterations and of immune system genes, including *TNFα*, and *SLC1A1*, in combination with cerebral immunopathological reactions to among others group A ß-hemolytic streptococcus infections^26^ and Bornea virus infections^27, 28^ in the onset (and expression) of OCD. This has led to the concept of ‘PANDAS' and ‘non-PANDAS' OCD.^29, 30, 31^ However, very little research has been performed on direct gene–gene interactions/pathways or on gene–environment interactions to better understand immunopathological pathways related to OCS. *MEF2B* is a protein-coding gene belonging to the DNA-binding protein family MADS/MEF2, that regulates gene expression, specifically in the smooth muscle tissue. Both *MEF2BNB* and *MEF2BNB-MEF2B* are closely related to *MEF2B*, and have mostly regulatory functions. Additional support for a relation between MEF2BNB and OCD comes from a gene-based analysis in a recently published GWAS of OCD^8^ where the gene was ranked 21st of the 21 567 genes tested (gene-based *P*=8.09E−04, not significant after correction for multiple testing). All four genes are overlapping, and span a region of 56302 bp located in the 19p13.11 cytogenetic band, on the short arm of chromosome 19. Perhaps the best interpretation for these results, therefore, is in the implication of this genomic location (19p13.11) as a susceptibility locus for OCS. Several SNP–trait associations have been linked to this locus. For example, rs1064395 (*NCAN*) has been reported as a susceptibility factor for bipolar disorder in a GWAS. The *NCAN* gene is located just 10095 bp at 5′ from *RFXANK*, and is one of the few genetic variants that has been genome-wide replicated as a risk factor in both bipolar disorder and schizophrenia.^32, 33^ In addition, a recent study focusing on the cortical thickness and folding in schizophrenia patients found evidence for association of the *NCAN* genetic variant in the occipital and prefrontal cortex.^34^ The SNP rs874628 (*MPV17L2* gene), located in this locus, was implicated in multiple sclerosis, an inflammatory disease with disruptions in the nervous system.^35^

Overall, our results, combined with previous genetic studies in OCD, suggest a possible role for the 19p13.11 region (MEF2BNB gene) in OCS. It might be of interest for future genetic studies to investigate this area in association with OCD into depth. Further, our data shows that well-phenotyped population cohorts could contribute to the understanding of the underlying architecture of common complex disorders such as OCS, and that these partly overlap with results from case–control studies. Therefore, future studies could benefit from combining case–control and population-based samples.

## Figures and Tables

**Table 1:** Subjects (including age and sex) entered per analysis

	N		*Sex (M/F (%))*		*Age (mean (s.d.))*	
	*PI-R-ABBR 2002*	*PI-R-ABBR 2008*	*PI-R-ABBR 2002*	*PI-R-ABBR 2008*	*PI-R-ABBR 2002*	*PI-R-ABBR 2008*
SEM (twins and sibs)	5780	9147	33.7/66.3	31.9/68.1	33.0(11.5)	34.7(14.6)
PRS (genotyped sample)	6931		35.7/64.3		42.8 (15.7)	
GCTA (genotyped sample)	6881		38.0/62.0		46.7 (15.4)	
GWAS (genotyped sample)	6931		35.7/64.3		42.8 (15.7)	

Abbreviations: GCTA, genome-wide complex trait analysis; GWAS, genome-wide association study; PI-R-ABBR, Padua Inventory Revised Abbreviated; PI-R-ABBR 2002, PI-R-ABBR collected in 2002; PI-R-ABBR 2008, PI-R-ABBR collected in 2008; PRS, polygenic score; SEM, structural equation modeling.

**Table 2:** Familial correlations estimated from maximum likelihood for OCS measured over two points in time

	*Twin correlation*	*Cross-twin—cross-time correlation*	*Retest stability (within person)*
MZ	0.41	0.36	
			0.63
DZ/siblings	0.13	0.11	

Abbreviations: DZ, dizygotic; MZ, monozygotic; OCS, obsessive–compulsive symptoms.

**Table 3:** Top associated variants in NTR-GWAS analysis

*SNP*	*CHR*	*BP(hg19)*	P*-value*	*A1/A2*	*BETA*	*Intragenic location*		
						*Left gene (kb)*		*Right gene (kb)*
rs8100480	19	19299079	2.56E−08	C/T	1.4095		*MEF2BNB*	
rs11671119	19	19286077	4.11E−07	C/T	1.2036		*MEF2BNB-MEF2B*	
rs4818048	21	40908952	4.24E−07	C/G	0.8956	*LOC729056 (7173)*		*B3GALT5(19417)*
rs4818050	21	40910600	6.37E−07	C/T	0.8725	*LOC729056 (8821)*		*B3GALT5(17769)*
rs77959192	21	40911027	7.36E−07	C/A	0.8707	*LOC729056 (9248)*		*B3GALT5(17342)*
rs4818049	21	40910464	1.03E−06	G/A	0.8557	*LOC729056 (8625)*		*B3GALT5(17965)*
rs77615161	21	40911050	1.30E−06	G/A	0.8541	*LOC729056 (9271)*		*B3GALT5(17365)*
rs17384439	4	96424680	1.59E−06	T/C	0.8323		*UNC5C*	
rs2837096	21	40978013	2.18E−06	G/A	0.747		*C21orf88*	
rs4818052	21	40912745	2.64E−06	A/G	0.8475	*LOC729056 (10966)*		*B3GALT5(15670)*
rs77460585	5	101123995	4.45E−06	G/A	0.8668	*LOC100420593*		*OR7H2P*
rs581043	3	62830115	5.26E−06	C/T	0.4358		*CADPS*	
rs999719	22	34264838	5.33E−06	T/A	0.4442		*LARGE*	
.	4	96399160	5.57E−06	R/D	0.7201			
rs74276709	21	40913995	5.85E−06	A/G	0.8226	*LOC729056*		*B3GALT5*
rs17024030	4	96399606	6.07E−06	G/A	0.7155		*UNC5C*	
rs9520326	13	107865442	6.37E−06	T/C	-0.4326		*FAM155A*	
rs79219884	21	40899981	6.40E−06	A/T	0.8467		*LOC729056*	
rs60588302	9	7900777	6.44E−06	C/T	1.1278	*C9orf123* (100971)		*TPRD* (413469)
rs11658311	17	17470526	6.50E−06	C/T	0.7719		*PEMT*	

Abbreviation: NTR-GWAS, Netherlands Twin Registry-genome-wide association study.

Single nucletotide polymorphisms (SNP) listed include the top 20 *P-*values for the GWAS results. Chromosome (Chr) and base pair (BP) position, based on hg19 build, are also given. The beta indicates the effect size, and the direction of the association is given by its positive or negative value. The location of each SNP is given in the last column; when located in non-intronic locus, the left and right closest flanking genes are additionally noted. A1 and A2 indicate the effect allele and the non-effect allele, respectively.

**Table 4:** List of all nominal significant genes (*α*=0.05)

*Gene*	*Chromosome*	*Start position*	*Length*	*Number of SNPs*	*Gene* P*-value*
RFXANK*	19	19303574	9104	10	5.60E−07
MEF2BNB*	19	19292684	10716	40	9.72E−07
MEF2BNB-MEF2B*	19	19256375	47025	73	1.29E−06
MEF2B*	19	19256375	24723	41	8.08E−06
*C21orf88*	21	40969074	15675	14	8.22E−05
*SAFB2*	19	5587009	35929	22	8.97E−05
*PEMT*	17	17408876	71903	73	1.02E−04
*SIAE*	11	12450568	40515	1	2.94E−04
*ATP5G1*	17	46970147	3085	18	3.47E−04
*CCL2*	17	32582295	1925	1	3.48E−04
*GOPC*	6	11788143	42273	17	3.61E−04
*TMEM63C*	14	77648101	77737	90	4.25E−04
*DKFZP434H168*	16	56226528	1909	1	4.51E−04
*UNC5C*	4	96083655	38670	43	5.20E−04
*UBE2Z*	17	46985730	20692	70	5.43E−04
*SMCR5*	17	17679999	2844	8	5.58E−04
*C19orf70*	19	5678432	2479	1	6.01E−04
*SNF8*	17	47007458	14696	59	6.99E−04
*SAFB*	19	5623045	45444	4	7.25E−04
*LOC101929154*	5	77180479	74441	20	7.27E−04
*MIR602*	9	14073287	98	2	7.34E−04
*MIR128-2*	3	35785967	84	1	7.85E−04
*NCAN*	19	19322781	40280	25	7.89E−04
*RPL36*	19	5690271	1407	3	8.49E−04
*CECR1*	22	17659679	20994	28	8.61E−04
*HSD11B1L*	19	5680775	7759	4	9.12E−04
*RP11-461O7.1*	16	56126898	98108	10	9.98E−04

Abbreviations: FDR, false discovery rate; GWAS, genome-wide association study; SNP, single nucleotide polymorphism.

Genes significant after FDR correction are depicted with a (*). For each gene, the chromosome, start position and respective gene length (in bp) are given. The number of SNPs from the GWAS within each gene are also present. The last column represent the calculated gene-based test *P*-value.
