# Supplementary material for: Obsessive–compulsive symptoms in a large population-based twin-family sample are predicted by clinically based polygenic scores and by genome-wide SNPs
Source: Transl Psychiatry. 2016 Feb 9;6(2):e731–. doi: 10.1038/tp.2015.223 (PMC4872426; doi:10.1038/tp.2015.223)
Supplement: supplementary Methods [file tp2015223x1.doc]

**Supplementary methods**

**Subjects entered into different genetic analyses**

For longitudinal heritability analysis we included a maximum of two twins and four siblings (two brothers and two sisters) per family. In total, for the PI-R-ABBR collected in 2002, 5.780 subjects were available, including 2223 monozygotic (MZ) twins (635M/1588F), 1207 dizygotic same sex (DZ-SS) twins (360M/847F), 955 dizygotic opposite sex (DZ-OS) twins (403M/552F)and 1395 siblings (552M/843F). For the PI-R-ABBR collected in 2008, a total of 9.147 subjects were available, including 3731 MZ twins (1017M/2714F), 2035 dizygotic same sex (DZ-SS) twins (623M/1412F), 1683 dizygotic opposite sex (DZ-OS) twins (646M/1037F) and 1698 siblings (629M/1069F).

**Participants and measures**

The PI-R-ABBR is derived from the Padua Inventory-Revised (PI-R), a widely used self-report inventory on OCS (1;2). The short version of the self-report instrument includes the subscales washing, checking, rumination, precision and impulses and has 12 items, which were chosen by selecting the 2 items per subscale that have had the highest factor loadings in a validation study (3) and an additional item for subscales on rumination and impulses (4;5). Each item is scored on a scale ranging from 0-4, with the OC symptom score per individual calculated as the total sum score (ranging from 0-48).

**Genotyping and imputation**

After DNA extraction and purification, genotyping was performed on multiple array platforms (AFFYMETRIX PERLEGEN 5.0, ILLUMINA 370, ILLUMINA 660, ILLUMINA OMNI EXPRESS 1M and AFFYMETRIX 6.0).Platform specific software (GENOTYPER, BEADSTUDIO and BIRDSEED) were used for genotype calling. Quality control was performed within and between chip platforms. First, individual SNP markers were lifted over to build 37 (HG19) of the Human reference genome, using the LIFTOVER tool (6). Second, SNPs that were not mapped at all, SNPs that had ambiguous locations, and SNPs that did not have matching or strand opposite alleles, were removed. Subsequently, the data were strand aligned with the 1000 Genomes phase 1 INTEGRATED RELEASE version 3 ALL panel of March 2012 (7). If a SNP still had mismatching alleles with the imputation reference set, if the allele frequencies differed more than 0.20 with the reference set, if the MAF was <1%, if the HWE P-value was <0.00001 or if the call rate was <95%, they were also removed. In addition, samples were excluded from the data if their expected sex did not match their genotyped sex, if the genotype missing rate was above 10% or if the Plink F inbreeding value was either >0.10 or <−0.10. After quality control, data of the individual chips were merged into a single dataset using the PLINK 1.07 software (8). Within the merged set, identity by descent (IBD) was calculated between all possible pairs of individuals and compared to the expected family structure of the NTR studies. Samples were removed when the data did not match the expected IBD sharing, or, alternatively, when potentially consistent with biographic data, corrections were made to the family structure. For DNA samples that were genotyped on multiple platforms we tested if the overlapping SNPs had a concordance rate above 99.0%. If not, data from these samples were removed. HWE and MAF SNP ﬁlters as well as reference allele frequency difference <0.20 checks were re-applied on the merged data. As a ﬁnal quality step prior to imputation SNPs with C/G and A/T allele combinations were removed if the MAF was between 0.35 and 0.50 to avoid wrong strand alignment. Imputation was performed using the two stage approach. Pre-imputation phasing and imputation of genotype platform speciﬁc SNPs was carried out using the MACH software (9). Subsequently, imputation of the reference set was carried out with Minimach. Prior to imputation the person with the highest SNP call rate within an MZ twin pair was selected. Post imputation, the resulting imputed genotypes were duplicated back to the co-twin in the data (10).

**SEM methods**

First, familial correlations were estimated. We tested for sex differences in variances and correlations, and for age regression on the OC symptom scores. Next, a bivariate model (with input of a 12 x 12 data matrix; 2 twins, 2 brothers and 2 sisters, with two measures) was fitted to the data to estimate the contribution of A, D and E variance components within and across time points. Parameter estimation was done by raw-data maximum likelihood, and comparison of submodels (model AE, assuming no dominance component) was done by means of likelihood-ratio tests, whose distribution follows a χ2 with degrees of freedom equal to the difference in the number of parameters of the two models. If the more restricted model better ﬁts the data, the more general model is dropped. The constrained model was deemed not signiﬁcantly worse than the previous model if the likelihood-ratio tests yielded a p value > 0.01. For both males and females, and for both PI-R-ABBR collected in 2002 and 2008, the distribution of the data was skewed (skewness_PI-R-ABBR 2002_ = 1.100; skewness_PI-R-ABBR 2008_ = 1.217). Therefore, data were square root transformed before genetic modeling.

**Estimations of variance explained by common SNPs (GCTA)**

From the complete 1000 genomes imputed data transformed to best guess Plink binary format SNPs were selected with a minimum imputation R^2^ quality metric of 0.80. In order to avoid explained and artificially increased GRM differences through differences between platforms and between subsamples, additional SNP quality control included evaluation of the SNP platform effect. This was done by defining individuals on a specific platform as cases and the remaining individuals as controls. Allelic association was calculated and SNPs were removed if allele frequencies were significantly different between platforms (p-value < 0.00001). Sex and age were included as covariates. The GRM was calculated per chromosome using GCTA and subsequently the 22 matrixes were merged into a single GRM based on 6.325.240 SNPs that remained in analysis. For the method proposed by Zaitlen et al (11), a second variance component is included in the model, that only considers closely related individuals for a certain predefined threshold of genetic relatedness (here defined only those with identity by descent values greater than 0.05). At the current sample size of 6881, the power to detect heritability of 0.14 is 86% (http://spark.rstudio.com/ctgg/gctaPower/).

**GWAS**

Imputed SNPs were analyzed in PLINK software (8). Genotype imputation uncertainty was accounted for by using allelic dosage (<http://pngu.mgh.harvard.edu/~purcell/plink/>). To correct for familial relatedness robust standard errors provided by the sandwich estimator in Plink (12) were computed. For quality control we filtered on the minor allele frequency (MAF) to ensure a minimum of 5 samples in the minor allele homozygous group, which was calculated as the sqrt(5/effective sample size)=sqrt(5/6931)=0.03. Further, we filtered on INFO=0.4-1.1, to get rid of SNPs with a bad imputation quality, and calculated a sample based Hardy-Weinberg Equilibrium (HWE) and only included SNPs with a HWE expected p-value>0.00001. At the threshold of α=5.00E-08, the required sample size for 80% power to detect a SNP at a minor allele frequency of 0.03, accounting for a proportion of total variance of 0.0025 is 16.950 unrelated individuals (http://pngu.mgh.harvard.edu/~purcell/gpc/). This indicates the low power of the study and the need for future Meta-analysis.

Reference List

(1) Sanavio E. Obsessions and compulsions: the Padua Inventory. Behav Res Ther 1988;26(2):169-77.

(2) van Oppen P. Obsessions and compulsions: dimensional structure, reliability, convergent and divergent validity of the Padua Inventory. Behav Res Ther 1992 Nov;30(6):631-7.

(3) van Oppen P, Hoekstra RJ, Emmelkamp PM. The structure of obsessive-compulsive symptoms. Behav Res Ther 1995 Jan;33(1):15-23.

(4) Cath DC, van Grootheest DS, Willemsen G, van Oppen P, Boomsma DI. Environmental factors in obsessive-compulsive behavior: evidence from discordant and concordant monozygotic twins. Behav Genet 2008 Mar;38(2):108-20.

(5) den Braber A, van 't Ent D, Cath DC, Wagner J, Boomsma DI, de Geus EJ. Brain activation during cognitive planning in twins discordant or concordant for obsessive-compulsive symptoms. Brain 2010 Oct;133(10):3123-40.

(6) Kuhn RM, Karolchik D, Zweig AS, Wang T, Smith KE, Rosenbloom KR, et al. The UCSC Genome Browser Database: update 2009. Nucleic Acids Res 2009 Jan;37(Database issue):D755-D761.

(7) Abecasis GR, Auton A, Brooks LD, DePristo MA, Durbin RM, Handsaker RE, et al. An integrated map of genetic variation from 1,092 human genomes. Nature 2012 Nov 1;491(7422):56-65.

(8) Purcell S, Neale B, Todd-Brown K, Thomas L, Ferreira MA, Bender D, et al. PLINK: a tool set for whole-genome association and population-based linkage analyses. Am J Hum Genet 2007 Sep;81(3):559-75.

(9) Li Y, Willer CJ, Ding J, Scheet P, Abecasis GR. MaCH: using sequence and genotype data to estimate haplotypes and unobserved genotypes. Genet Epidemiol 2010 Dec;34(8):816-34.

(10) Nivard MG, Mbarek H, Hottenga JJ, Smit JH, Jansen R, Penninx BW, et al. Further confirmation of the association between anxiety and CTNND2: replication in humans. Genes Brain Behav 2014 Feb;13(2):195-201.

(11) Zaitlen N, Kraft P, Patterson N, Pasaniuc B, Bhatia G, Pollack S, et al. Using extended genealogy to estimate components of heritability for 23 quantitative and dichotomous traits. PLoS Genet 2013 May;9(5):e1003520.

(12) Minica CC, Dolan CV, Kampert MM, Boomsma DI, Vink JM. Sandwich corrected standard errors in family-based genome-wide association studies. Eur J Hum Genet 2014 Jun 11.
