# Supplementary material for: Obsessive–compulsive symptoms in a large population-based twin-family sample are predicted by clinically based polygenic scores and by genome-wide SNPs
Source: Transl Psychiatry. 2016 Feb 9;6(2):e731–. doi: 10.1038/tp.2015.223 (PMC4872426; doi:10.1038/tp.2015.223)
Supplement: Supplementary Figure Legends [file tp2015223x2.doc]

**Supplementary legends**

**Figures**

***Supplementary figure 1.*** Distribution of OC symptom scores, before transformation of the data, in both PI-R-ABBR collected in 2002 (left), and 2008 (right).

***Supplementary figure 2.*** *Proportion of variance in height, as measured in the NTR sample, explained by polygenic scores (PRS) obtained from European case-control sample by Stewart et al. 2013,* *with a range of 15 statistical cutoffs for SNP inclusion in the score (PRS1; p<0.00001, PRS2; p<0.0001, PRS3; p<0.001, PRS4; p<0.01, PRS5; p<0.05, PRS6; p<0.1, PRS7; p<0.2, PRS8; p<0.3, PRS9; p<0.4, PRS10; p<0.5, PRS11; p<0.6, PRS12; p<0.7, PRS13; p<0.8, PRS14; p<0.9, PRS15; p<=1).*

***Supplementary figure 3.*** *Fisher’s exact test heatmap plot for clinical OCD p-values (P2) conditioned on OC symptom scores p-values(P1).*

***Supplementary figure 4.*** *Quantile--quantile (QQ) plots of observed versus expected -log (P) statistics*

***Supplementary figure 5.*** *Regional association plot for the top region identified in the GWAS. The top associated SNP (rs8100480, purple dot at the center) is depicted in a genomic window of 800 Kb (hg19). P-values are given, as well as linkage disequillibrium (LD) strength (r^2^; data from the 1000 genomes project European samples) between the sentinel SNP and its flanking markers; the strength of the association is illustrated by the colour of the dots for neighbouring markers (dark blue – low LD, red – high LD). The blue lines indicate the estimated recombination rate (cM per Mb).*

***Supplementary figure 6.*** *Quantile-quantile plot for the association analysis.*

***Supplementary figure 7.*** *Manhattan plot for the full gene association analysis.*

**Tables**

***Supplementary table 1.*** *Extension of table 3. Listed are all the SNPs with a P-value < 10-6 for the GWAS results. Allele frequency and INFO represent additional information on the frequency of the minor allele and the imputation info score, respectively. Chromosome (Chr) and base pair position (BP), based on hg19 build, are also given. The beta indicates the effect size, and the direction of the association is given by its positive or negative value. The location of each SNP is given in the last column; when located in non-intronic locus, the left and right closest flanking genes are additionally noted. A1 and A2 indicate the effect allele and the non-effect allele, respectively.*

***Supplementary table 2.*** *Abbreviations: SNP, single nucleotide polymorphism; CHR, chromosome; BP, base pair position; IOCDF-GC, The international OCD foundation Genetics Collabarative; MA, minor allele in IOCDF-GC meta-analyses; NA, not applicable. SNPs listed are strongest associated GWAS variants in combined trio–case-control samples reported in Table 1 of Stewart et al. 2013. Directions of effects (IOCDF-GC: DIR and OCS-NTR: DIR) are given based on the A1 in the IOCDF analyses. For the IOCDF-GC GWAS, directions are given for all study samples (EU, AJ, SA, trios).*

***Supplementary table 3.*** *Abbreviations: SNP, single nucleotide polymorphism; CHR, chromosome; BP, base pair position; OCGAS, The OCD Collaborative Genetics Association Study; MA, minor allele in OCGAS meta-analyses; NA, not applicable. SNPs listed are strongest associated GWAS variants reported in Table 1 of Mattheisen et al. 2014. Directions of effects (OCGAS: DIR and OCS-NTR: DIR) are given based on the A1 in the OCGAS analyses).*
