# Supplementary figures and images for: Obsessive–compulsive symptoms in a large population-based twin-family sample are predicted by clinically based polygenic scores and by genome-wide SNPs
Source: Transl Psychiatry. 2016 Feb 9;6(2):e731–. doi: 10.1038/tp.2015.223 (PMC4872426; doi:10.1038/tp.2015.223)

***Supplementary figure 1***


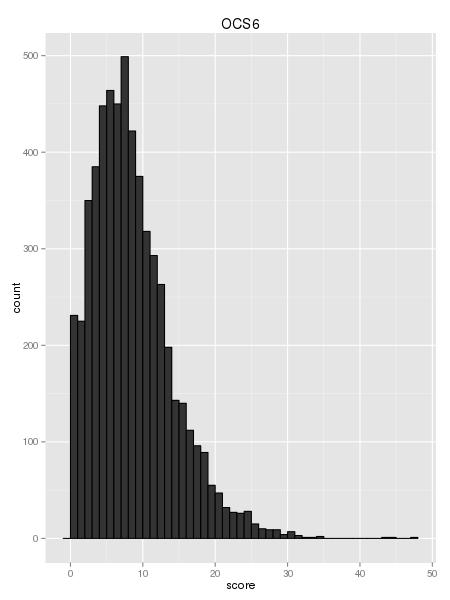

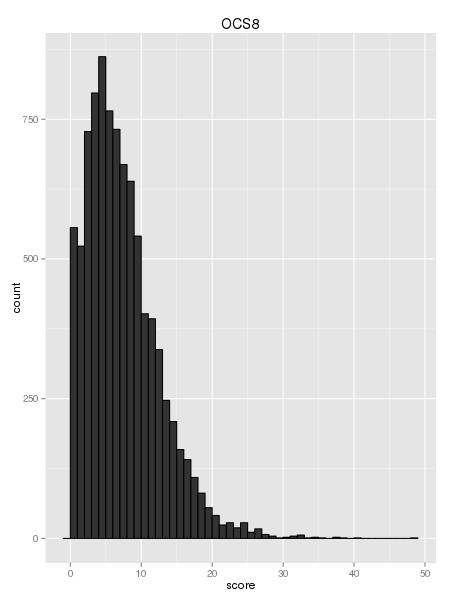

Supplement: Supplementary Figure 1 [file tp2015223x3.doc]

***Supplementary figure 2***


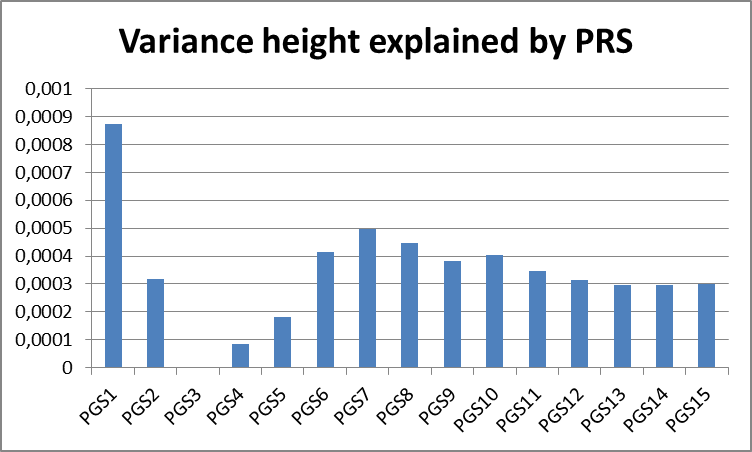


| **PRS** | **PRS cutoff** |
| --- | --- |
| 1 | 0 - 0.00001 |
| 2 | 0 - 0.0001 |
| 3 | 0 - 0.001 |
| 4 | 0 - 0.01 |
| 5 | 0 - 0.05 |
| 6 | 0 - 0.1 |
| 7 | 0 - 0.2 |
| 8 | 0 - 0.3 |
| 9 | 0 - 0.4 |
| 10 | 0 - 0.5 |
| 11 | 0 - 0.6 |
| 12 | 0 - 0.7 |
| 13 | 0 - 0.8 |
| 14 | 0 - 0.9 |
| 15 | 0 - 1 |

Supplement: Supplementary Figure 2 [file tp2015223x4.doc]

***Supplementary figure 3***


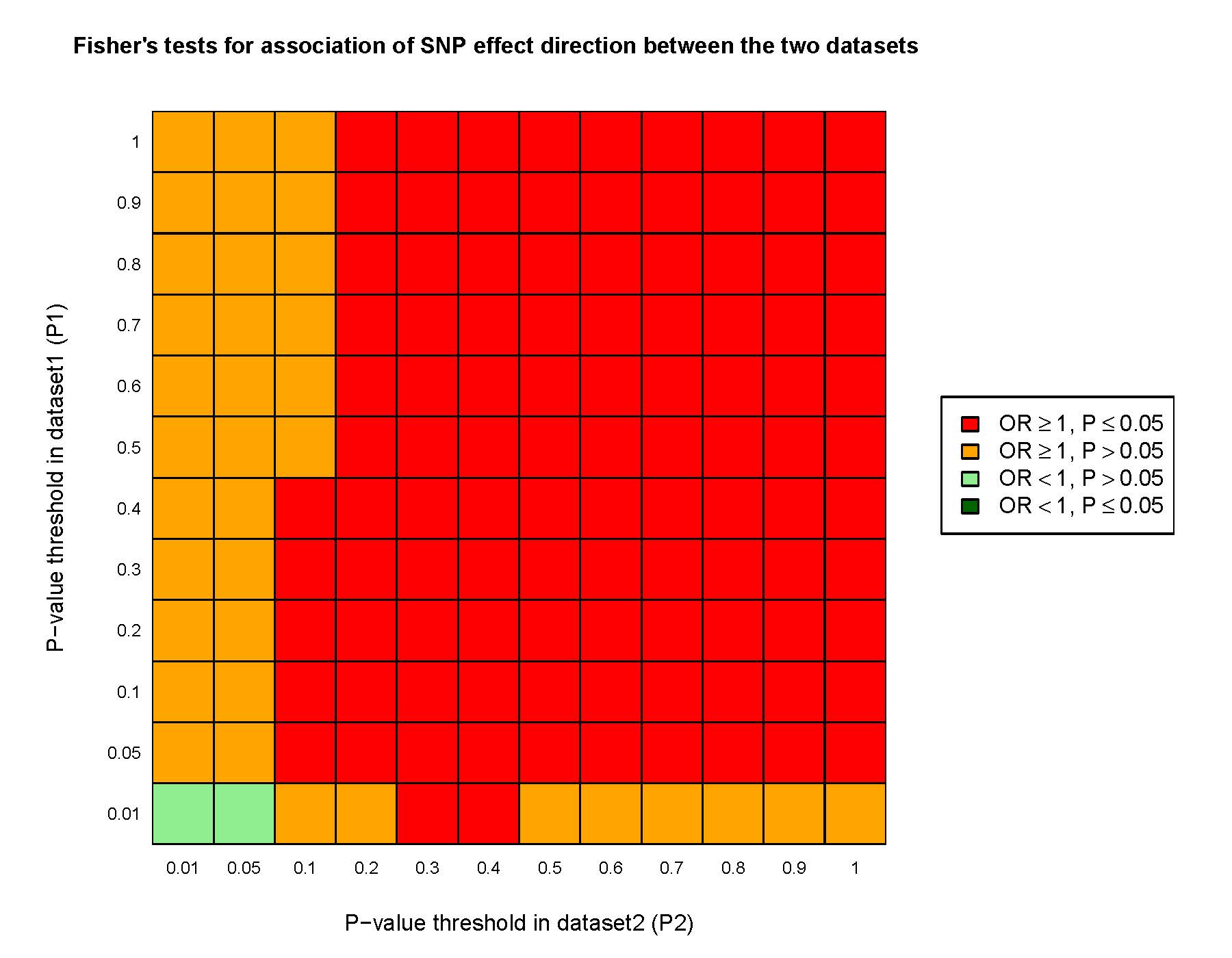

Supplement: Supplementary Figure 3 [file tp2015223x5.doc]

***Supplementary figure 4***


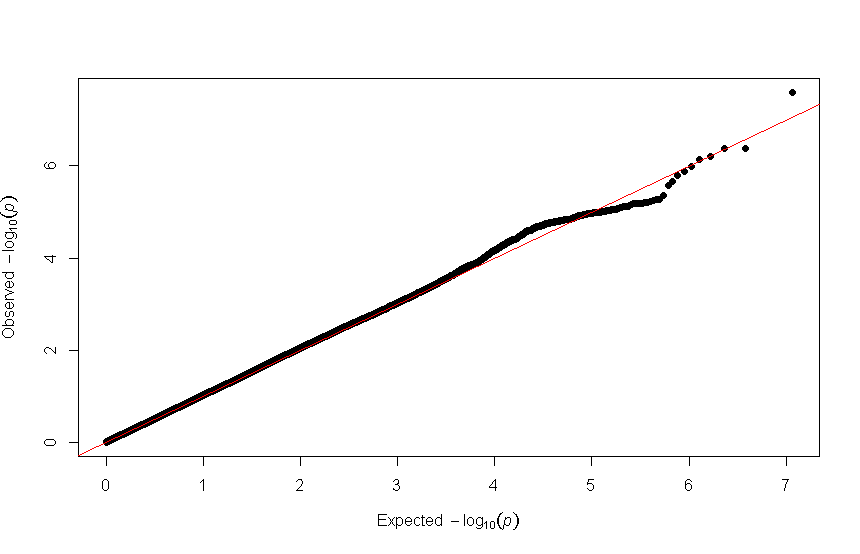

Supplement: Supplementary Figure 4 [file tp2015223x6.doc]

***Supplementary figure 5***


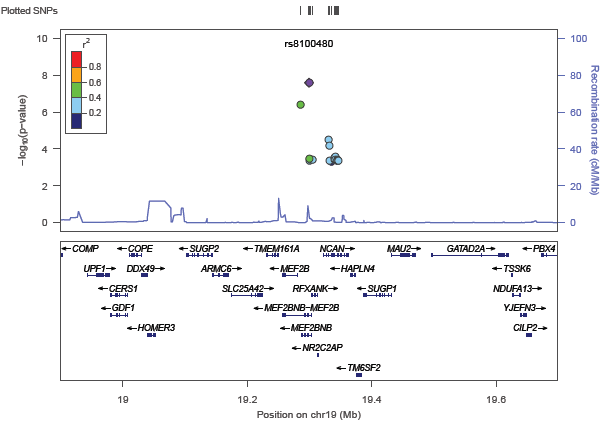

Supplement: Supplementary Figure 5 [file tp2015223x7.doc]

***Supplementary figure 6***


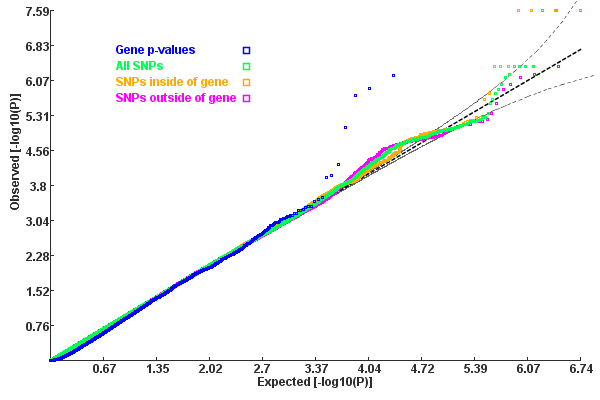

Supplement: Supplementary Figure 6 [file tp2015223x8.doc]

***Supplementary figure 7***


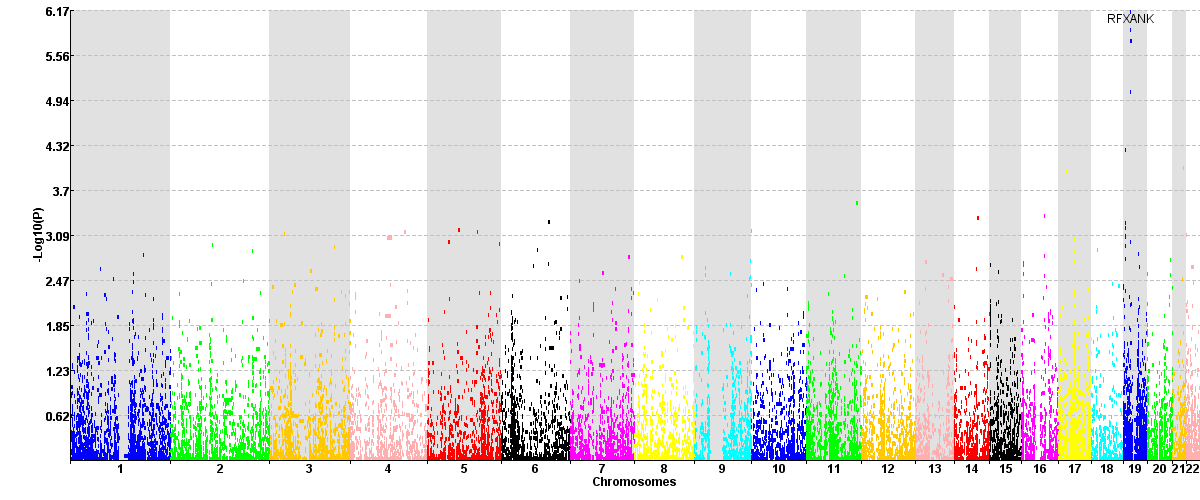

Supplement: Supplementary Figure 7 [file tp2015223x9.doc]
