# Supplementary material for: Obsessive–compulsive symptoms in a large population-based twin-family sample are predicted by clinically based polygenic scores and by genome-wide SNPs
Source: Transl Psychiatry. 2016 Feb 9;6(2):e731–. doi: 10.1038/tp.2015.223 (PMC4872426; doi:10.1038/tp.2015.223)
Supplement: Supplementary Tables [file tp2015223x10.doc]

| **Supplementary table 1.** Top associated variants in NTR-GWAS analysis | | | | | | | | | | |
| --- | --- | --- | --- | --- | --- | --- | --- | --- | --- | --- |
| **SNP** | **CHR** | **BP(hg19)** | **P-value** | **A1/A2** | **BETA** | **Allele frequency** | **INFO** |  | **Intragenic location** |  |
|  |  |  |  |  |  |  |  | **Left gene(kb)** |  | **Right gene(kb)** |
| rs8100480 | 19 | 19299079 | 2.56E-08 | C/T | 1.4095 | 0.0367 | 0.6877 |  | *MEF2BNB* |  |
| rs11671119 | 19 | 19286077 | 4.11E-07 | C/T | 1.2036 | 0.0302 | 0.9816 |  | *MEF2BNB-MEF2B* |  |
| rs4818048 | 21 | 40908952 | 4.24E-07 | C/G | 0.8956 | 0.0601 | 0.9606 | *LOC729056 (7173)* |  | *B3GALT5(19417)* |
| rs4818050 | 21 | 40910600 | 6.37E-07 | C/T | 0.8725 | 0.0597 | 0.9789 | *LOC729056 (8821)* |  | *B3GALT5(17769)* |
| rs77959192 | 21 | 40911027 | 7.36E-07 | C/A | 0.8707 | 0.0595 | 0.9782 | *LOC729056 (9248)* |  | *B3GALT5(17342)* |
| rs4818049 | 21 | 40910464 | 1.03E-06 | G/A | 0.8557 | 0.0609 | 0.9734 | *LOC729056 (8625)* |  | *B3GALT5(17965)* |
| rs77615161 | 21 | 40911050 | 1.30E-06 | G/A | 0.8541 | 0.0603 | 0.9714 | *LOC729056 (9271)* |  | *B3GALT5(17365)* |
| rs17384439 | 4 | 96424680 | 1.59E-06 | T/C | 0.8323 | 0.0589 | 0.896 |  | *UNC5C* |  |
| rs2837096 | 21 | 40978013 | 2.18E-06 | G/A | 0.747 | 0.0765 | 0.9688 |  | *C21orf88* |  |
| rs4818052 | 21 | 40912745 | 2.64E-06 | A/G | 0.8475 | 0.0581 | 0.9723 | *LOC729056 (10966)* |  | *B3GALT5(15670)* |
| rs77460585 | 5 | 101123995 | 4.45E-06 | G/A | 0.8668 | 0.0539 | 0.9539 | *LOC100420593* |  | *OR7H2P* |
| rs581043 | 3 | 62830115 | 5.26E-06 | C/T | 0.4358 | 0.3043 | 0.9916 |  | *CADPS* |  |
| rs999719 | 22 | 34264838 | 5.33E-06 | T/A | 0.4442 | 0.3063 | 10.007 |  | *LARGE* |  |
| . | 4 | 96399160 | 5.57E-06 | R/D | 0.7201 | 0.0682 | 0.9803 |  |  |  |
| rs74276709 | 21 | 40913995 | 5.85E-06 | A/G | 0.8226 | 0.056 | 0.9907 | *LOC729056* |  | *B3GALT5* |
| rs17024030 | 4 | 96399606 | 6.07E-06 | G/A | 0.7155 | 0.0681 | 0.9851 |  | *UNC5C* |  |
| rs9520326 | 13 | 107865442 | 6.37E-06 | T/C | -0.4326 | 0.4301 | 0.9852 |  | *FAM155A* |  |
| rs79219884 | 21 | 40899981 | 6.40E-06 | A/T | 0.8467 | 0.0547 | 0.9694 |  | *LOC729056* |  |
| rs60588302 | 9 | 7900777 | 6.44E-06 | C/T | 11.278 | 0.0278 | 0.9002 | *C9orf123* (100971) |  | *TPRD* (413469) |
| rs11658311 | 17 | 17470526 | 6.50E-06 | C/T | 0.7719 | 0.0622 | 10.119 |  | *PEMT* |  |
| rs182187683 | 21 | 40908159 | 6.52E-06 | A/G | 0.8927 | 0.0507 | 0.9494 | *LOC729056* (6380) |  | *B3GALT5* (20210) |
| rs186307979 | 21 | 40908160 | 6.53E-06 | G/T | 0.8926 | 0.0507 | 0.9492 | *LOC729056* (6381) |  | *B3GALT5* (20209) |
| rs113123495 | 19 | 5587540 | 6.78E-06 | C/T | 10.582 | 0.0349 | 0.7935 |  | *SAFB2* |  |
| rs9619381 | 22 | 34262097 | 7.20E-06 | G/T | 0.4396 | 0.3058 | 0.999 |  | *LARGE* |  |
| rs10815705 | 9 | 7902402 | 7.58E-06 | T/G | 11.251 | 0.0275 | 0.9184 | *C9orf123* (102596) |  | *PTPRD* (411844) |
| rs75494728 | 1 | 90440967 | 7.69E-06 | G/A | 0.9241 | 0.1016 | 0.47 | *LRRC8D* (38976) |  | *ZNF326* (19711) |
| rs142967832 | 21 | 40900470 | 7.72E-06 | T/C | 0.8391 | 0.0545 | 0.9729 |  | *LOC729056* |  |
| rs75477292 | 21 | 40900608 | 7.78E-06 | G/A | 0.8389 | 0.0545 | 0.973 |  | *LOC729056* |  |
| rs57277948 | 17 | 17481317 | 7.96E-06 | T/C | 0.856 | 0.062 | 0.8334 |  | *PEMT* |  |
| rs79874412 | 21 | 40900861 | 8.18E-06 | G/A | 0.8376 | 0.0544 | 0.9735 |  | *LOC729056* |  |
| rs117114519 | 21 | 40901172 | 8.30E-06 | T/C | 0.8372 | 0.0544 | 0.9739 |  | *LOC729056* |  |
| rs117465668 | 21 | 40901183 | 8.36E-06 | C/G | 0.8371 | 0.0544 | 0.9739 |  | *LOC729056* |  |
| . | 17 | 17471374 | 8.74E-06 | R/I | 0.7628 | 0.0623 | 10.104 |  | *EEF1A1P43* |  |
| rs7332896 | 13 | 107865698 | 8.82E-06 | C/T | -0.4269 | 0.4297 | 0.9897 |  | *FAM155A* |  |
| rs74813064 | 21 | 40901563 | 9.01E-06 | T/C | 0.8358 | 0.0542 | 0.9745 |  | *LOC729056* |  |
| rs67366981 | 14 | 77703964 | 9.01E-06 | T/C | -0.6535 | 0.1253 | 10.126 |  | *TMEM63C* |  |
| rs67130018 | 14 | 77703280 | 9.00E-06 | C/T | -0.6815 | 0.1205 | 0.9691 |  | *TMEM63C* |  |
| rs9904004 | 17 | 17476791 | 9.06E-06 | A/G | 0.7702 | 0.0618 | 10.096 |  | *PEMT* |  |
| rs7394339 | 10 | 85120682 | 9.15E-06 | G/A | -0.6012 | 0.1486 | 0.9544 | *MARK2P15* (46475) |  | *HMGN2P8* (720405) |
| rs1997543 | 21 | 40974288 | 9.23E-06 | G/C | 0.698 | 0.0734 | 10.069 |  | *C21orf88* |  |
| rs12627674 | 21 | 40901786 | 9.27E-06 | G/C | 0.8366 | 0.0539 | 0.9745 | *LOC729056* (7) |  | *B3GALT5*(26583) |
| rs8007762 | 14 | 77708465 | 9.62E-06 | A/C | -0.6602 | 0.1287 | 0.9759 |  | *TMEM63C* |  |
| rs12627733 | 21 | 40902206 | 9.74E-06 | C/T | 0.8386 | 0.0534 | 0.9741 | *LOC729056* (427) |  | *B3GALT5* (26163) |
| rs698737 | 8 | 117222665 | 9.75E-06 | G/C | 11.281 | 0.033 | 0.8179 | *TRPS1* (509366) |  | *EIF3H* (434390) |
| rs750095 | 13 | 107868758 | 9.86E-06 | G/C | -0.4258 | 0.3919 | 0.992 |  | *FAM155A* |  |
| rs17798894 | 3 | 9061714 | 9.90E-06 | T/G | 0.4585 | 0.2303 | 10.133 |  | *SRGAP3* |  |

| **Supplementary table 2.** Follow-up of IOCDF-GC GWAS results | | | | | | | | | | | | | | |
| --- | --- | --- | --- | --- | --- | --- | --- | --- | --- | --- | --- | --- | --- | --- |
| **MARKERINFO** | | |  | **IOCDF-GC** | | |  | **OCS-NTR** | |  | **Intragenic location** | | | |
| **SNP** | **CHR** | **BP (hg19)** |  | **P-Value** | **A1/A2** | **DIR** |  | **P-Value** | **DIR** |  | **Left gene** |  | **Right gene** | |
| rs11898020 | 2 | 144282078 |  | 2.56E-04 | A/G | +-+- |  | 0.3457 | - |  |  | ARHGAP15 | |  |
| rs10165908 | 2 | 158315629 |  | 0.0169 | C/T | +++- |  | 0.227 | - |  | CYTIP |  | | ACVR1C |
| rs1838733 | 5 | 58533392 |  | 3.82E-05 | T/C | ---- |  | 0.4355 | - |  |  | PDE4D | |  |
| rs26728 | 5 | 106946056 |  | 1.01E-04 | T/C | -++- |  | 0.2454 | - |  |  | EFNA5 | |  |
| **rs4868342** | **5** | **173504522** |  | **3.20E-05** | **C/T** | **++++** |  | **0.0049** | **+** |  |  | **HMP19** | |  |
| rs9499708 | 6 | 104445367 |  | 2.96E-06 | C/T | --+- |  | NA | NA |  | GRIK2 |  | | HACE1 |
| rs2205748 | 6 | 104462555 |  | 8.52E-06 | G/A | ++++ |  | NA | NA |  | GRIK2 |  | | HACE1 |
| rs182320 | 6 | 130073291 |  | 2.25E-05 | C/T | ++++ |  | 0.8009 | + |  | ARHGAP18 |  | | C6orf191 |
| rs6531002 | 8 | 12722703 |  | 0.0067 | T/C | -++- |  | NA | NA |  | LONRF1 |  | | KIAA1456 |
| rs11611761 | 12 | 33025612 |  | 0.115 | A/C | --+- |  | 0.0746 | + |  |  | PKP2 | |  |
| rs297941 | 12 | 50319086 |  | 4.99E-07 | G/A | ---- |  | NA | NA |  | FAIM2 |  | | AQP2 |
| rs9652236 | 13 | 72688774 |  | 5.14E-06 | T/G | ++++ |  | 0.4476 | - |  | DACH1 |  | | MZT1 |
| rs11081062 | 18 | 3662879 |  | 2.92E-05 | T/C | +0++ |  | 0.6318 | + |  |  | DLGAP1 | |  |
| rs11663827 | 18 | 3663631 |  | 2.31E-05 | A/G | ++++ |  | 0.5986 | + |  |  | DLGAP1 | |  |
| rs485186 | 19 | 49207206 |  | 9.94E-06 | G/A | ++++ |  | 0.3297 | - |  |  | FUT2 | |  |
| rs6131295 | 20 | 11996267 |  | 3.63E-05 | G/A | ---+ |  | 0.7904 | - |  | BTBD3 |  | | SPTLC3 |

| **Supplementary table 3.** Follow-up of OCGAS-GWAS results | | | | | | | | | | | | | | |
| --- | --- | --- | --- | --- | --- | --- | --- | --- | --- | --- | --- | --- | --- | --- |
| **MARKERINFO** | | |  | **OCGAS** | | |  | **OCS-NTR** | |  | **Intragenic location** | | | |
| **SNP** | **CHR** | **BP (hg19)** |  | **P-Value** | **A1/A2** | **DIR** |  | **P-Value** | **DIR** |  | **Left gene** |  | | **Right gene** |
| rs4401971 | 9 | 11890045 |  | 4.13E-07 | A/G | - |  | 0.1437 | + |  | LOC646114 |  | LOC100049717 | |
| rs6876547 | 5 | 25572301 |  | 1.76E-06 | G/T | + |  | 0.0825 | + |  | CDH10 |  | MSNL1 | |
| rs13437953 | 7 | 54313171 |  | 9.69E-06 | A/C | - |  | 0.6638 | + |  | FLJ45974 |  | LOC222005 | |
| rs6452234 | 5 | 24922789 |  | 1.13E-05 | A/G | - |  | NA | NA |  | CDH10 |  | MSNL1 | |
| rs10149510 | 14 | 24039559 |  | 1.49E-05 | T/C | + |  | NA | NA |  |  | JPH4 |  | |
| rs7462051 | 8 | 80266553 |  | 1.69E-05 | A/G | + |  | 0.6567 | + |  | IL7 |  | LOC100128963 | |
| rs1544352 | 16 | 19713882 |  | 1.94E-05 | C/T | + |  | NA | NA |  | C16orf62 |  | C16orf88 | |
| rs1532154 | 4 | 157279663 |  | 2.22E-05 | C/T | - |  | 0.4974 | - |  | FTHP2 |  | hCG_1814936 | |
| rs509876 | 11 | 79811268 |  | 2.29E-05 | G/A | + |  | 0.1965 | - |  | LOC646112 |  | LOC729790 | |
| rs10392 | 20 | 37550935 |  | 2.87E-05 | A/G | - |  | 0.0484 | - |  |  | PPP1R16B |  | |
| rs2821204 | 9 | 11683901 |  | 2.89E-05 | G/A | + |  | 0.8537 | + |  | LOC646114 |  | LOC100049717 | |
| rs10882583 | 10 | 97109019 |  | 3.56E-05 | A/G | - |  | 0.1149 | - |  |  | SORBS1 |  | |
| rs2278144 | 8 | 25634077 |  | 3.94E-05 | T/C | - |  | 0.7461 | - |  | CDCA2 |  | EBF2 | |
| rs8026755 | 15 | 37247722 |  | 4.03E-05 | A/C | - |  | 0.7806 | + |  |  | MEIS2 |  | |
| rs838209 | 3 | 176352763 |  | 4.14E-05 | C/T | - |  | 0.1190 | - |  | LOC730168 |  | TBL1XR1 | |
| rs17735629 | 5 | 168653502 |  | 5.03E-05 | C/T | + |  | 0.6468 | + |  |  | SLIT3 |  | |
| rs7003102 | 8 | 126303493 |  | 5.14E-05 | G/A | + |  | 0.0598 | - |  |  | NSMCE2 |  | |
| rs12547343 | 8 | 26833198 |  | 5.38E-05 | A/C | - |  | 0.4138 | - |  | LOC100127897 |  | LOC100132229 | |
| rs7593878 | 2 | 44358504 |  | 5.44E-05 | C/A | - |  | 0.9034 | + |  | LRPPRC |  | PPM1B | |
| rs16867406 | 2 | 182189405 |  | 6.10E-05 | T/C | + |  | 0.8464 | - |  | LOC729026 |  | LOC100127923 | |
| rs9541148 | 13 | 35095884 |  | 6.73E-05 | C/T | - |  | NA | NA |  | LOC100130499 |  | LOC100129452 | |
| rs1671253 | 18 | 50040561 |  | 7.04E-05 | T/C | - |  | 0.9944 | + |  |  | DCC |  | |
| rs973714 | 6 | 133578733 |  | 7.44E-05 | A/G | + |  | 0.9983 | + |  |  | EYA4 |  | |
| rs3902042 | 10 | 17065067 |  | 7.71E-05 | T/C | + |  | NA | NA |  |  | CUBN |  | |
| rs8120171 | 20 | 50712059 |  | 7.85E-05 | T/C | - |  | NA | NA |  |  | ZFP64 |  | |
| rs16851014 | 4 | 75216365 |  | 7.89E-05 | A/G | - |  | 0.2436 | - |  | EPGN |  | EREG | |
| rs9845643 | 3 | 179531062 |  | 8.58E-05 | G/A | - |  | 0.4279 | - |  |  | PEX5L |  | |
| rs11265908 | 9 | 92077132 |  | 8.60E-05 | C/T | - |  | 0.3481 | - |  |  | SEMA4D |  | |
| rs16997877 | 20 | 16533162 |  | 8.68E-05 | C/T | + |  | 0.7431 | - |  |  | KIF16B |  | |
| rs2183738 | 9 | 71211200 |  | 8.73E-05 | G/A | + |  | 0.5192 | + |  | C9orf71 |  | LOC347097 | |
| rs12096987 | 1 | 185201061 |  | 8.87E-05 | C/T | + |  | 0.6838 | + |  |  | C1orf26 |  | |
| rs7005206 | 8 | 130620813 |  | 9.13E-05 | A/G | - |  | 0.7417 | + |  | LOC100129525 |  | LOC100130376 | |
